# Diversity of the surface microbiome of canopy-forming brown macroalgae (Fucales) in the northern Adriatic

**DOI:** 10.1128/spectrum.02204-24

**Published:** 2025-04-16

**Authors:** Neža Orel, Ana Lokovšek, Martina Orlando-Bonaca, Tinkara Tinta

**Affiliations:** 1 Marine Biology Station Piran, National Institute of Biology 354430 , Piran, Slovenia; 2 Jožef Stefan International Postgraduate School, Ljubljana, Slovenia; DePauw University, Greencastle, Indiana, USA

**Keywords:** *Gongolaria barbata*, Fucales, macroalgae-associated microbiomes, host-microbiome, 16S rRNA amplicon sequencing, anthropogenically impacted coastal ecosystems

## Abstract

Canopy-forming brown macroalgae (Fucales) offer numerous key ecosystem services in Mediterranean coastal areas. However, anthropogenic pressures and climate change have significantly impacted their habitats, leading to an extensive population decline. Interactions between algae and microbiota are a major ecological aspect, yet they represent a significant knowledge gap. In our baseline study, we describe the diversity and host specificity of the microbiome of two genetically identical but morphologically distinct populations of *Gongolaria barbata* from anthropogenically impacted northern Adriatic Sea. Our preliminary results showed that the microbiomes of *G. barbata* exhibited low host specificity, with 75% of the algae-associated amplicon sequence variants (ASVs) being part of the core coastal ecosystem microbiome. However, microbiomes of specific algal parts, ambient seawater, and sediment differed significantly in terms of alpha diversity and composition. In contrast, the holdfast and axis show higher similarity with sediment microbiomes, indicating potential horizontal transmission pathways. Microbiomes associated with deciduous parts of morphologically distinct *G. barbata* populations showed no difference in alpha diversity and composition. In contrast, higher variation in alpha diversity and lower sequence proportion of shared ASVs were observed in the holdfast and axis of the two distinct populations. Our observational study provides valuable new insights and baseline for future hypothesis-driven research on the interactions between algae and associated microbiota—a knowledge gap that needs to be addressed in the future for better understanding of the ecological and evolutionary dynamics of coastal ecosystems.

**IMPORTANCE:**

Our study focuses on the microbiomes of canopy-forming brown macroalgae from the Fucales order, essential habitat builders in Mediterranean coastal areas. These habitats, offering key ecosystem services, face significant declines due to anthropogenic pressures and climate change. We used next-generation 16S rRNA amplicon sequencing to reveal novel insights into the diversity and host specificity of *Gongolaria barbata* populations in impacted ecosystems. Our findings suggest environmental factors influence the structure of the algae microbiome, with potential recruitment from adjacent sediment communities. This research enhances the understanding of marine ecosystems’ ecological and evolutionary dynamics, providing valuable insights for conservation and management efforts.

## INTRODUCTION

Canopy-forming Fucales (Phaeophyta) are dominant habitat builders along rocky shores of the Mediterranean Sea (1, 2). In particular, species of the genus *Cystoseira sensu lato*, lately subdivided into the three genera *Cystoseira*, *Ericaria*, and *Gongolaria* (3), which can grow from intertidal to upper-circalittoral bottoms, are building dense forests that are among the most productive communities in the Mediterranean coastal area (4), providing several key ecosystem services (5–10).

Due to interplaying anthropogenic stressors (e.g., coastal constructions, local pollution sources, and sediment resuspension) and climate change (e.g., increase of seawater temperature), *Cystoseira s.l*. populations have gradually disappeared in many coastal areas in the Mediterranean Sea (4, 11, 12). A sharp decline of fucoids has also been reported for the anthropogenically impacted coastal zone of the Gulf of Trieste, northern Adriatic Sea (11–13), where genetically identical yet morphologically different populations can be found at different locations along the Slovenian coast (14). When studying the effects of climate change and/or anthropogenic pressures on eukaryotic organisms, we often overlook a major ecological aspect: the interactions with associated microbiota. Yet, according to the “meta-organism” concept, any multicellular organism is composed of the macroscopic host in a synergistic interaction with microorganisms (15, 16). This concept postulates that microbes are an essential part of the host’s phenotype and hence should be considered when addressing evolutionary and ecological aspects of the host (17, 18). The importance of the microbiota for the host’s fitness, health, reproduction, behavior, population dynamics, interactions with the environment, survival, and decay are increasingly recognized for a wide diversity of meta-organisms (16). The microbiome of diverse algae is quite well studied, yet being host-species-specific (19, 20), we are far from capturing the entire algae diversity (e.g., less abundant, endangered, difficult to access) and functional potential of algae-associated microbiota. The algal microbiome can have beneficial and detrimental effects on the host; it has been shown that it modulates algal growth, morphogenesis, reproduction, and chemical defense against other micro- and macro-fouler (21, 22). Yet, the underlying mechanisms of algae-microbes associations are largely unknown.

Despite numerous studies on the rich macrofauna and flora associated with *Cystoseira s.l.*, there is little knowledge about its associated microbiota (23–25). These studies collectively underline the significance of *Cystoseira s.l.*-associated microbiomes in shaping host health, stress resilience, and potential restoration strategies, while also pointing to gaps in understanding their functional roles and interactions. Among these studies, only Mancuso et al. (23) provided some insights into host specificity toward individual algae parts, while none of these studies explicitly addressed host specificity at the level of different *Cystoseria s.l.* populations. To preserve their populations and the ecosystem services they provide in the ocean of change, the associated microbiota as crucial components of *Cystoseira s.l.* metaorganism need to be further explored. Building up on previous studies, our objectives were to (i) further characterize the composition of the *Gongolaria barbata* microbiome, (ii) determine host specificity, specifically toward individual algae part and host population, and (iii) to study differences in the microbiome of two genetically identical populations of *G. barbata* in the northern Adriatic, which show considerable morphological differences and could therefore also harbor significantly different microbiomes. We employed a high-throughput next-generation sequencing approach to provide insights into microbial diversity at unprecedented detail, allowing for the comprehensive analysis of microbiota composition and variation among different algal parts and populations.

## MATERIALS AND METHODS

### Study area and sampling campaign

**Fig 1:**
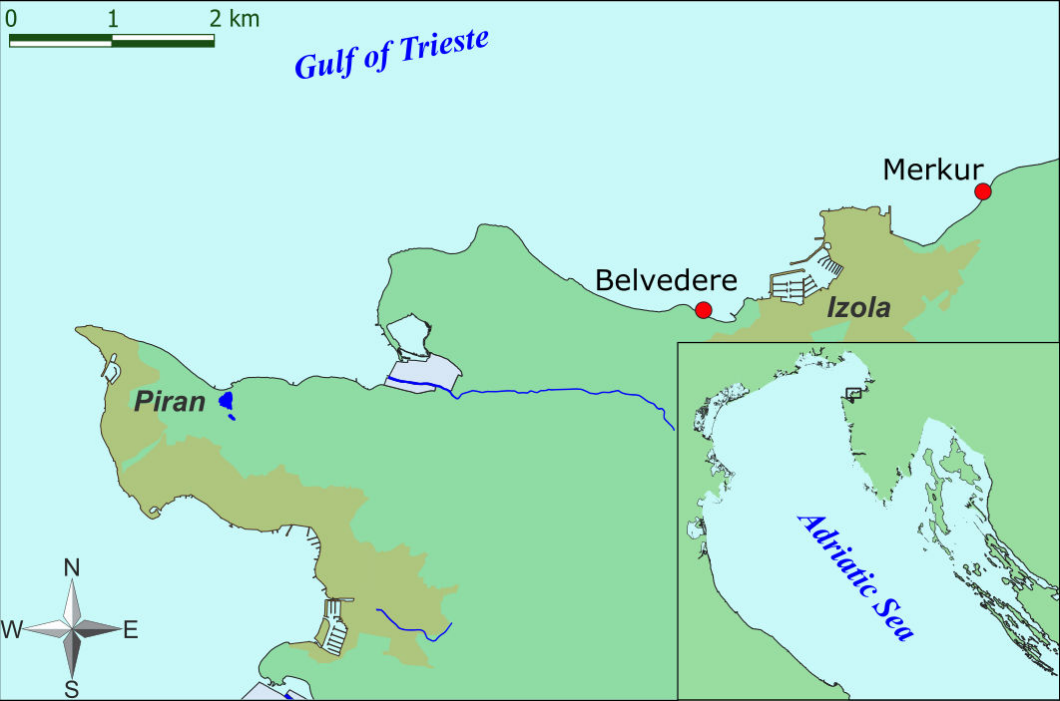
Map of the study area showing the two sampling sites (Belvedere and Merkur) of *G. barbata* populations, near Izola.

**Fig 2:**
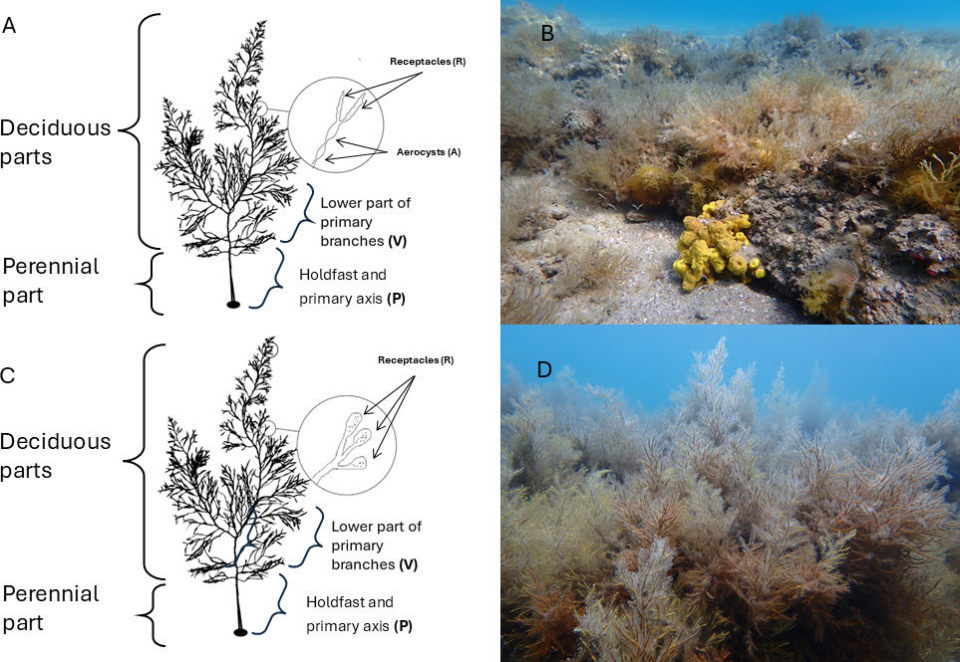
Phenotypically distinct populations of *Gongolaria barbata* from the two sampling sites. (**A)** Schematic drawing of a thallus from the Belvedere population, characterized by the absence of aerocysts and smaller receptacles. (**B)** Photography of the Belvedere population, characterized by lower growth.** (C)** Schematic drawing of a thallus from the Merkur population, featuring several aerocysts and longer receptacles. (**D)** Photography of the Merkur population, with taller thalli reaching up to 1 m.

The Gulf of Trieste is a shallow, semi-enclosed bay in the northernmost part of the Adriatic Sea. For many decades, the coastal area has been affected by various anthropogenic impacts such as construction, intensive fishing, sewage discharges, and mariculture (26). Thalli of *G. barbata* were collected by SCUBA divers in April 2022 from two sampling sites (Fig. 1), selected according to Lokovšek et al. (27). The first site, Belvedere, is characterized by a dense population of *G. barbata* (Fig. 2B). The apical fronds of these thalli had short, simple, and cylindrical receptacles that were sparsely mucronate and had few or no aerocysts. The algae grow on sandstone boulders that are surrounded by sandy substrate. The second site, Merkur, is characterized by dense populations of *G. barbata* and *Cystoseira compressa* (Fig. 2D). The apical fronds of these thalli had long fusiform or mucronate receptacles, with numerous single or concatenated aerocysts. The algae grow on flysch terraces positioned between areas with bare sand. Terraces are often covered by sediments from flysch crumbling. The area is exposed to freshwater inputs from the Rižana and Badaševica rivers (28); therefore, a high resuspension of sediments is present, with consequent lower water transparency.

The Merkur site was sampled on 4 April 2022, and the Belvedere site on 5 April 2022. At each site, three whole thalli of *G. barbata* were collected by scraping beneath their holdfast on boulders, at approximately 2 m depth. The samples were stored in zip-lock plastic bags to protect them from external contaminants and preserve the integrity of the associated microbiome. Additionally, seawater samples were collected ~0.5 m above the thalli (one 1.5 L sample per location), in pre-acid washed, Milli-Q rinsed plastic bottles, and sediment samples were taken with small sterile corers (one 200 mL corer per location), very close to the algal holdfast (approximately 2 cm–5 cm). All samples were immediately transported (within 30 min) to the laboratory, where they were further processed. During transportation, the sampled material was stored in a portable refrigerator.

In the laboratory, all thalli individual body parts were divided into subsamples (~10 g for each part in 50 mL falcon tube): (i) holdfast and lower part of the cauloid (primary axis) (P), (ii) lower part of primary branches (V), (iii) aerocysts (A), and (iv) receptacles (R) (Fig. 2A and C). For analysis of ambient microbiomes, 1 L of ambient seawater samples were filtered onto 0.2 µm polyethersulfone filters (PALL Life Sciences), and ambient sediment samples were stored in sterile 50 mL tubes. All the samples were then stored in sterile cryo tubes at −80°C until DNA extraction. For chemical analysis, inorganic nutrients, and dissolved organic matter analysis, samples were processed as described under “Chemistry,” below.

### Analysis of algae-associated and ambient microbiome using 16S rRNA amplicon sequencing

DNA was extracted from the filters with the DNeasy PowerWater Kit (Qiagen). Two hundred fifty milligrams of sediment was used for DNA extraction with a DNeasy PowerSoil Kit (Qiagen). One hundred milligrams of algae was used for DNA extraction with the DNeasy Plant Pro Kit (Qiagen). Amplification and sequencing were performed by Microsynth AG (Balgach, Switzerland). Primers 515F (5′-GTG YCA GCM GCC GCG GTA A-3′) (29) and 805R (5′-GGA CTA CNV GGG TWT CTA AT-3′) (30) were used for amplification of the V4 region of bacterial 16S rRNA gene. The amplicons were sequenced on the Illumina Miseq platform (2 × 250 bp paired-end). Sequencing generated a data set of 25 samples with 9,309,075 read pairs (Table S2).

Bioinformatic and statistical analyses were conducted in R (v.4.2.2). Paired-end reads were trimmed, denoised, and merged using the “DADA2” package (31). Forward and reverse reads were quality trimmed to 150 bp at a maximum expected error rate of 2. Sample inference was done in “pool” mode to resolve also rare amplicon sequence variants (ASVs). After merging paired-end reads, chimeras were removed with the method “pooled”. Only ASVs occurring at least twice and with a length between 252 and 256 bp were kept for further analyses, resulting in 347,887 ± 135,941 merged sequences per sample (Table S2). Taxonomy was assigned using the SILVA database (SSU 138.1) (32).

Further taxonomic filtration and analysis of the microbial community described in this paragraph were performed using “phyloseq” (33). ASVs assigned to Eukaryota, chloroplast, and Mitochondria or not assigned at the phylum level were excluded. The “iNEXT” (34) package was used to make rarefaction curves for each sample. The number of observed ASVs, Chao1, Shannon, and inverse Simpson indices were calculated through the “phyloseq” package to describe alpha diversity. A test for a significant difference in alpha diversity indexes was performed by one-way analysis of variance (ANOVA). The test was selected based on a previous analysis of normality using the Shapiro-Wilk method. The presence of a significant difference between sample groups is indicated with a significance level of at least *P* < 0.05. *Post hoc* analyses were performed using the Tukey test. Phyla with a total abundance of less than 35 were removed for further analyses. Bacterial community composition was analyzed using weighted principal coordinates analysis (PCoA) based on Bray-Curtis distances calculated from relative abundance data. Permutational analysis of variance (PERMANOVA) was performed using the adonis2 function in the R package “vegan” (35) to test for significant differences between groups of samples, with 999 permutations. *Post hoc* pairwise PERMANOVA comparisons were conducted for algal body parts and ambient samples using the “pairwiseAdonis” package (36). Shared ASVs were visualized with “venn” (v.1.1) (37).

### Chemistry

#### Dissolved organic carbon (DOC) and nitrogen

Samples for DOC and total dissolved nitrogen (TDN) analyses were filtered through combusted Whatman GF/F (~0.8 µm pore size) filters using acid-, Milli-Q water rinsed and a combusted glass filtration system. DOC and TDN analyses were performed by a high-temperature catalytic method using a Shimadzu TOC-L analyzer equipped with a total nitrogen unit (38). The calibration for non-purgeable organic carbon was done with potassium phthalate, and for TDN, potassium nitrate was used. The results were validated with Deep-Sea Reference water for DOC (CRM Program, Hansell Lab). The precision of the method, expressed as relative standard deviation, was <2%.

#### Dissolved inorganic nutrients

Dissolved inorganic nitrogen (NH_4_^+^, NO_2_^-^, NO_3_^-^) and dissolved inorganic phosphorus (PO_4_^3-^) concentrations were determined spectrophotometrically by segmented flow analysis (QuAAtro, Seal Analytical) following standard methods (39). The validation and accuracy of the results were checked with reference material (KANSO CO., LTD.) before and after sample analyses. The quality control is performed annually by participating in an inter-calibration program (QUASIMEME Laboratory Performance Study).

### Graphic design

Graphs were plotted in R Studio (40) with the “ggplot2” package (41). A graphical abstract was created with BioRender.com. The layout was adapted from “Ocean Layout,” by BioRender.com (2024), retrieved from https://app.biorender.com/biorender-templates. All final figures were edited using the vector graphics editor Inkscape v.1.1 (42). The map in Fig. 1 was made in ArcGIS and Fig. 2 in Adobe Illustrator and Microsoft Office PowerPoint.

## RESULTS

### Diversity differences between algal-associated and ambient microbial communities

We observed richness from 308 to 9,742 bacterial ASVs per sample (Table S2). Rarefaction curves reached a plateau in all of the examined communities (Fig. S1). Thus, our sequencing depth represents most of the bacterioplankton diversity (34). However, two samples of R microbiome from the Merkur sampling site (A1_R_IZ and A3_R_IZ) were removed from further analysis since only a low number of reads were obtained for these two samples (1,622 and 2,404, respectively).

**Fig 3:**
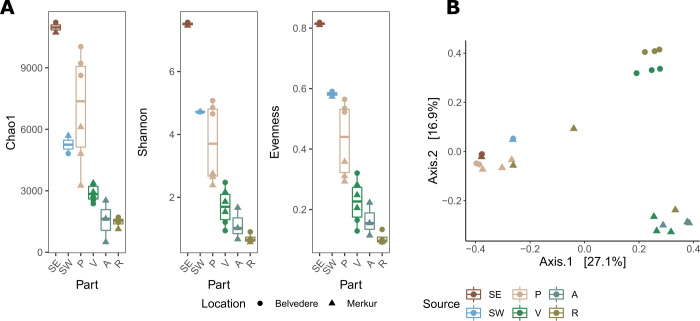
(A) Alpha diversity indices (Chao1 richness estimator, Shannon diversity index, and species evenness) of samples from distinct sources (different algal parts [**P, V, A, R**], seawater [SW], sediment). (**B)** Weighted PCoA based on Bray-Curtis distances of bacterial community composition. Each point represents a sample, colored by algal part or ambient source and shaped by sampling location.

There was significant variation among samples from distinct sources (different algal parts, seawater, sediment) for all alpha diversity indices (ANOVA test, Chao1: *F*_5,16_ = 20.69, *P* < 0.0001; Shannon: *F*_5,16_ = 35.64, *P* < 0.0001; Evenness: *F*_5,16_ = 35.54, *P* < 0.0001). Total species richness (Chao1) and Shannon diversity index of ambient sediment communities were significantly higher than that of ambient seawater assemblage (Tukey’s honestly significant difference [HSD], adjusted *P* < 0.05; Table S4, Fig. 3A). Bacterial communities associated with algae were overall characterized with significantly lower richness (Tukey’s HSD for V, A, and R, adjusted *P* < 0.0001), Shannon diversity index (Tukey’s HSD for V, A, and R, adjusted *P* < 0.0001; for P, adjusted *P* < 0.001), and evenness (Tukey’s HSD for V, A, and R, adjusted *P* < 0.0001; for P, adjusted *P* < 0.001) than that of the adjacent sediment, except for communities associated with holdfast and axis (P), which resembled species richness of sediment assemblages (Tukey’s HSD; Table S4, Fig. 3A). Algae-associated communities also exhibited significantly lower Shannon diversity index and evenness than ambient seawater assemblage (Tukey’s HSD for R, adjusted *P* < 0.001; for V and R, adjusted *P* < 0.01), except for communities associated with holdfast and axis (P) (Tukey’s HSD; Table S4, Fig. 3A). At the same time, there was no statistical difference in total species richness between ambient seawater- and algae-associated communities (Tukey’s HSD; Table S4, Fig. 3A). Within the algae microbiome, alpha diversity indices revealed no significant difference among communities associated with V, R, and A (Tukey’s HSD; Table S4, Fig. 3A). On the other hand, these communities exhibited significantly lower richness, Shannon diversity index, and evenness than communities associated with holdfast and axis (P) (Tukey’s HSD for all diversity indices: for R, adjusted *P* < 0.001; for V and A, adjusted *P* < 0.01) (Table S4, Fig. 3A). Alpha diversity indices revealed no differences between bacterial communities sampled at the two locations, except for species richness (ANOVA test, *F*_5,16_ = 20.69, *P* < 0.05; Table S3). On the other hand, after grouping by different algal parts, ANOVA showed that communities associated with holdfast and axis (P) were significantly different at the two locations in terms of species richness, Shannon diversity index, and evenness (Fig. 3A, Table S5).

Weighted PCoA based on Bray-Curtis distance revealed significant variation in bacterial community composition based on isolation source (algal body part and different ambient samples; PERMANOVA test, *R*^2^ = 0.55, *P* < 0.001) and spatial variation by sampling location (PERMANOVA test, *R*^2^ = 0.09, *P* < 0.001) (Fig. 3B). *Post hoc* pairwise PERMANOVA tests showed that communities associated with the holdfast and axis (P) significantly differed from all other algae-associated and seawater communities (*R*^2^ = 0.47 for A, *R*^2^ = 0.29 for R, adjusted *P* < 0.01; *R*^2^ = 0.38 for V, adjusted *P* < 0.001). These communities also differed from sediment and seawater communities, but with a lower significance level (*R*^2^ = 0.33 for sediment and *R*^2^ = 0.46 for seawater, adjusted *P* < 0.05) (Table S6).

**Fig 4:**
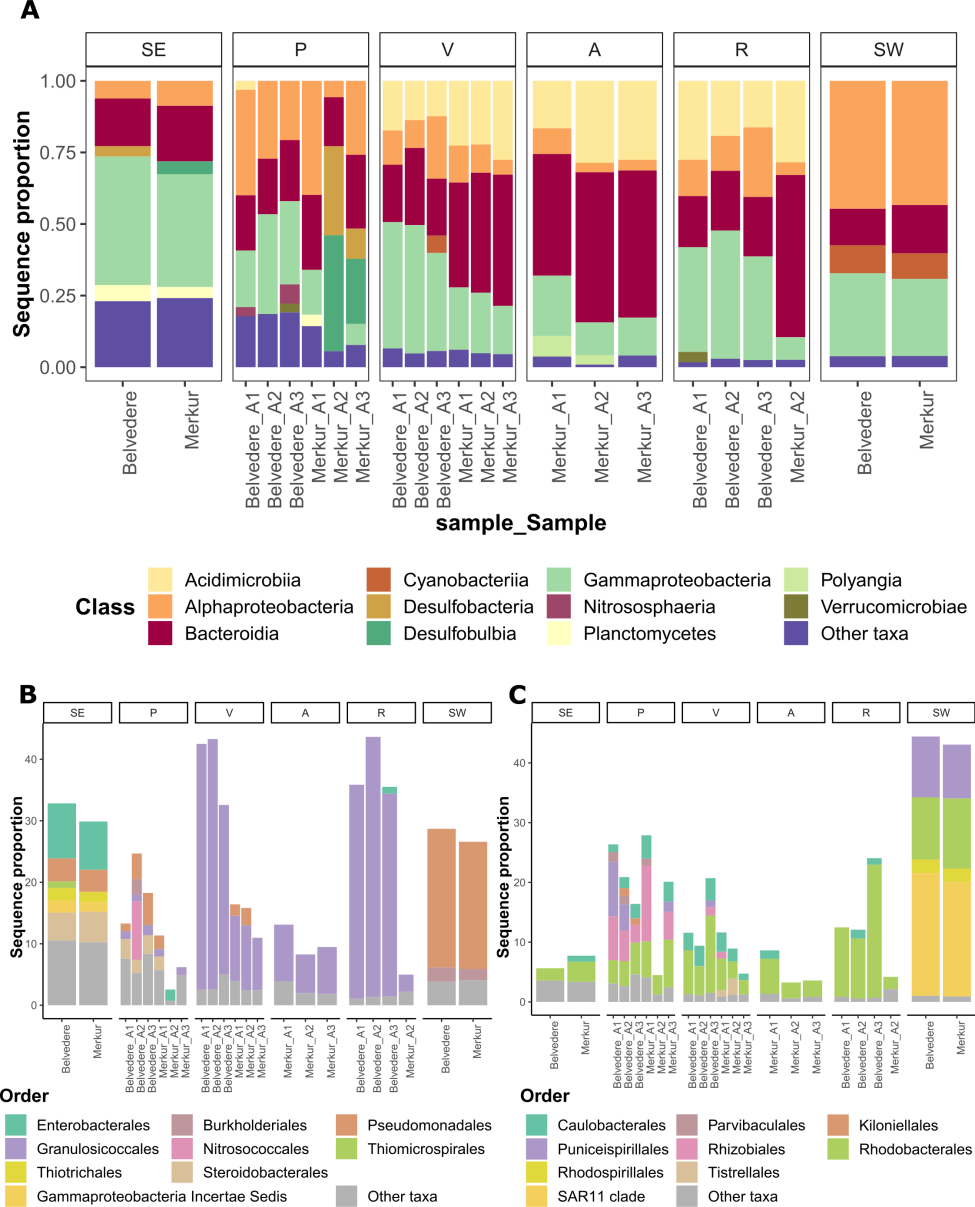
Sequence proportion of dominant bacterial genera associated with different algal parts (**P, V, A, R**), ambient seawater (SW), and sediment (SE) microbiomes in two different locations (Belvedere, Merkur). (**A)** Class-level bacterial community structure (classes showing a sequence proportion <3% are grouped as “other taxa”). (**B)** Order-level composition of Gammaproteobacterial populations (orders showing a sequence proportion <1% are grouped as “other taxa”). (**C)** Order-level composition of Alphaproteobacterial populations (orders showing a sequence proportion <1% are grouped as “other taxa”).

Taxonomic composition of algae-associated microbial communities differed from adjacent sediment and seawater communities. The most profound difference between algae-associated and ambient microbial communities was a higher relative abundance of Acidimicrobiia (within which 88.1%–100% were affiliated with the *Microtrichaceae* family) in algae-associated communities, representing 12.3%–28.6% of sequence proportions in A-, R-, and V-associated communities, while less than ~2% in ambient microbial communities (Fig. 4A). Sequence proportion represents the proportion of sequences belonging to a specific taxon (e.g. species, family, order, etc.) relative to the total number of sequences in the sample. Gammaproteobacteria were detected in all samples in our study, representing 26.8 to 43.9% of sequence proportions in ambient assemblage and 2.7%–44.8% in algae-associated communities (Fig. 4A). However, while the sediment and holdfast and axis (P) Gammaproteobacterial population was very diverse, the V, R, and A were predominated by the Granulosicoccales (Fig. 4B), more precisely *Granulosicoccaceae* family (56.3%–97.1% of Gammaproteobacteria population, Table S8). On the other hand, we could not detect *Granulosicoccaceae* in sediment or seawater assemblage (<0.1% of sequence proportion, Table S8). Bacteroidia were present in ambient and algae-associated communities, where they represented between 12.7 and 56.6% of sequence proportions, respectively, and were predominated by the *Flavobacteriaceae* family (51.7%–97.8% of the Bacteroidia population, Table S8), while Cytophagales and Bacteroidales were detected mainly in sediment and holdfast and axis (P) samples. Likewise, Desulfobacteria, Desulfobulbia, and Planctomycetes were more abundant in sediment and holdfast and axis (P) samples as compared to other algae parts and seawater, in particular at the Merkur site. *Nitrososphaera* was predominantly detected in holdfast and axis (P) samples (0.5–6.6 of sequence proportion), while Polyangia was mainly in aerocysts (2.6%–7% of sequence proportion). Ambient seawater communities were dominated by Alphaproteobacteria (44.6%–43.3% of sequence proportion, predominated by SAR11, followed by Rhodobacterales and Puniceispirillales) (Fig. 4C). On the other hand, this bacterial class represented only a minor part of alga-associated microbiomes (<24.3% of sequence proportion, predominantly affiliated with Rhodobacterales), except for the holdfast and axis (P), where Alphaproteobacteria represented 39.7–25.8 of the community (within which Rhodobacterales and Rhizobiales contributed equal or larger share compared to other algal parts) (Fig. 4C). Within Alphaproteobacterial populations Puniceispirillales, Kiloniellales, and Parvibaculales were unique to holdfast and axis (P), however representing only a minor percentage of total P-associated microbiome (Fig. 4C). Cyanobacteria were almost exclusively found in ambient seawater, where they represented around 10% (9.7%–8.9%) of the sequence proportion (Fig. 4A) and were dominated by Synechococcales, while they represented less than 6% of algae-associated and sediment communities, predominantly by Cyanobacteriales.

### Microbiome of *G. barbata*

Determination of the similarity of microbial communities associated with different algal parts, ambient seawater, and sediment was performed based on analysis of the presence/absence of ASV (Fig. S2). Core microbiome, i.e., ASVs detected in all samples of our study set, comprised 545 ASVs out of 13,723 ASVs recruited from all samples in total (Fig. S2A). Nevertheless, these core microbiome ASVs represented a major part of the bacterial community (74.2%–93.9% of sequence proportion) on deciduous parts (i.e. V, A, and R) implying a low level of specificity of the microbiota associated with algae (Fig. S3). Holdfast and axis (P) microbiomes comprise a lesser extent of the core ASVs (22.0%–58.6%, Fig. S3). The number of unique ASVs to the algae microbiome (i.e., not found in seawater or sediment) and shared among all parts of the algae was low (43) for the whole algae and was even lower for deciduous parts (6) (Fig. S2A). There were 1,082 ASVs unique to holdfast and axis (P) (mainly belonged to classes Bacteroidia, Alphaproteobacteria, Gammaproteobacteria and Planctomycetes), but only 17 ASVs to V (mainly Alphaproteobacteria, Bacteroidia, Gracilibacteria), 9 for A (mainly Bacteroidia) and 1 for R (Gammaproteobacteria) (Fig. 2A, Fig. S4). The holdfast and axis (P) and sediment samples shared the highest number of unique ASVs (3,487) (Fig. S2A), representing 14.3%–16.5% and 1.7%–8.9% of sequence proportion for sediment and holdfast and axis (P), respectively (Fig. S5). Among sediment and holdfast and axis (P)-specific ASVs, there were representatives of Gammaproteobacteria, Bacteroidia, Alphaproteobacteria, and Planctomycetes (Fig. S4).

When examining the two sampling locations individually, more differences become apparent. The Belvedere sampling site was characterized by a smaller number of ASVs unique to deciduous parts (76 for A, V, and R combined out of 13,007 ASVs in the Belvedere sampling site), as compared to the Merkur location (290 unique for A, V, and R combined out of 12,230 ASVs in Merkur sampling site) (Fig. S2B). About 5% of ASVs were shared between deciduous parts (A, V, and R combined) and holdfast and axis (P) at each sampling location, while there were more ASVs unique to holdfast and axis (P) at location Belvedere (1,940) as compared to Merkur (822) (Fig. S2B). There was a high number of ASVs shared between holdfast and axis (P) and sediment at both sampling locations; 3,848 and 1,696 at Belvedere and Merkur, respectively. (Fig. S2B).

When comparing the two sampling locations, 72.3% of sediment ASVs and 50.3% of holdfast and axis (P) ASVs were shared between both locations (Table S7), representing 59.6%–86.1% of sequence proportion in P samples (Fig. S6). Among microbial groups associated with holdfast and axis (P) at both sampling locations were *Desulfocapsaceae, Desulfosarcinaceae, Flavobacteriaceae, Rhodobacteraceae*, etc. (Fig. S6). At the same time, only 43.34% of deciduous parts ASVs were shared between the two locations (Table S7), representing 86.7%–97.6% of sequence proportion in deciduous parts samples (Fig. S6). Among microbial groups associated with algal deciduous parts at both sampling locations were *Flavobacteriaceae*, *Granulosicoccaceae*, *Microtrichaceae*, etc. (Fig. S7). When comparing specific deciduous parts separately, 40.9% and 11.1% of ASVs were shared between locations for primary branches and receptacles, respectively (Table S7).

## DISCUSSION

### Morphological plasticity in algal hosts is not dictating microbiome composition

The present study represents the first attempt to characterize in detail the microbiome associated with the two phenotypically distinct populations of the canopy-forming brown alga *G. barbata* in the anthropogenically impacted coastal zone of the northern Adriatic Sea. In this area, the species exhibits considerable morphological plasticity, with the peak of fertility between March and May (14, 44, 45). After molecular analyses, which revealed no genetic differences (14), the population at the Merkur site was attributed to the morphotype of *G. barbata* f. *hoppei* (elongated fronds and numerous oval to fusiform aerocysts), while the population from the Belvedere site can be considered as *G. barbata* f. *turneri* (short fronds and absent or very rare aerocysts) (Fig. 2). Although the two sites along the Slovenian coast are close to each other, our preliminary analysis suggests that they differ in the chemical characteristics of ambient seawater (Table S1). However, the sample size would need to be increased in the future to support our preliminary findings. Nevertheless, the measured concentration of organic and inorganic nutrients was within the range previously reported for the Gulf of Trieste (46, 47) (with slightly higher inorganic nutrient concentrations [in particular, NH_4_^+^ and NO_3_^-^], but lower DOC and TDN measured at the Belvedere site). The Merkur site is characterized by high sedimentation and suspension rates, resulting in increased water turbidity (48). In contrast, the Belvedere site is exposed to less anthropogenic pressure as it is located at the beginning of a still pristine coastline. Consequently, we can speculate that the two populations might be adapted to different sedimentation rates, salinity, and turbidity, which could potentially affect the length of the branches and the presence of aerocysts. However, additional analysis of environmental parameters at the two sites would need to be conducted in the future. Nevertheless, alpha diversity indices revealed no significant differences between bacterial communities associated with algae or ambient samples at the two locations, except for holdfast and axis, which differed in terms of species richness, Shannon diversity, and evenness (Fig. 3A, Table S5), suggesting a low degree of host specificity. Hence, based on our preliminary results, the two genetically identical populations of *G. barbata* in the northern Adriatic, which show considerable morphological differences, harbor similar microbiomes. Nevertheless, the deciduous parts from two locations shared 43.3% of ASVs, which accounted for the majority of sequence read proportions in these samples (86.7%–97.6%). In contrast, the holdfast and axis shared 50.3% of ASVs, representing a slightly smaller portion of their respective communities (59.6%–86.1%). This suggests that sediment may have a greater influence on the structure of the algal microbiome compared to the surrounding seawater. However, note that our study lacks chemical analysis of the sediment, and we did not conduct specific experiments to further investigate this aspect. Therefore, we cannot draw any conclusions about the impact of sediment chemistry on the microbiomes associated with algal holdfast and axis parts. On the other hand, when inspecting the deciduous part separately, we observed that primary branches from two sampling locations shared ~40% of ASVs, while receptacles shared only 11%, suggesting increasing environmental selective pressure, such as exposure to light, nutrient availability, or hydrodynamic forces, as algae grow higher up in the water column. At the same time, we acknowledge that our current study cannot separate environmental selective pressure from intrinsic factors of algae (e.g., tissue biochemical composition or physiology of different algal parts) as the drivers of observed microbial differences. Additional chemical, microbiological, and environmental analyses are required to investigate the influence of environmental selective pressures and algal intrinsic factors on microbiome composition. Indeed, it has been shown that macroalgae surfaces are rapidly colonized with bacteria, potentially recruited via horizontal transmission from surrounding seawater (49), forming dense surface biofilms (20, 43, 50), probably representing the first interface interacting with the surrounding system. It has been previously suggested that epiphytic bacteria colonizing algal tissues respond rapidly to fluctuations in environmental factors (24, 51).

### Microbiomes of different algal parts exhibit different levels of host specificity

Our preliminary results imply a low level of host specificity for the microbiota associated with *G. barbata,* as 75% of algae-associated ASVs were also detected in the core microbiome in our entire data set (i.e., ASVs detected in all sequenced samples from ambient seawater, sediment, and all algae thalli parts) (Fig. S2A and S3). Nevertheless, microbiomes associated with deciduous algae parts (i.e., primary branches, receptacles, and aerocysts) were characterized by significantly lower alpha diversity measures than ambient seawater and sediment microbiomes (Fig. 3A). These results are in line with the findings of previous studies, which suggested that epiphytic bacteria, particularly growing on Fucales, differ significantly from the microbiome on abiotic surfaces and in the surrounding water (50, 52). Compared to the algal thalli surfaces, the sediment and seawater generally had a slightly higher bacterial diversity, confirming earlier research postulating that algae actively select their associated microbiota (53).

On the other hand, there was no significant difference between species richness of microbiomes associated with holdfast and axis and ambient microbiomes. Additionally, our results show a high number of ASVs shared between holdfast and axis and sediment microbiota, at both sampling locations (Fig. S2A and S5). Based on this, one could speculate a horizontal mode of microbiome transmission (i.e., acquisition of microbiome from the environment and not from the parent–progeny relationship) from the sediment community (acting as seed source) to the algae holdfast and axis body parts. Even more so, when comparing the two locations, the potential source of microbial seeds was highly similar, with ~70% shared ASVs between sediment microbiomes, resulting in ~50% similarities between holdfast and axis microbiomes between the two locations. Furthermore, among sampled algal body parts, we observed the highest number of unique ASVs in holdfast and axis samples (1,082 out of 10,754 ASVs, representing 0.5%–4.5% of sequence proportions). Hence, this does suggest a certain level of host specificity of microbiota associated with algae holdfast and axis parts. We hypothesize that the algal-associated microbiota is acquired (e.g., via horizontal transmission) from sediment communities during early developmental stages, such as the holdfast and primary axis, which serve as anchors and are in direct contact with the sediment. This hypothesis will be addressed in future studies. Later, during the growth of the algae in the water column toward the light, the associated microbiota is subjected to greater selection pressure, resulting in significantly different microbial communities being associated with deciduous algal parts, characterized by lower alpha diversity (Fig. 3A). This again implies that this part of the algal thalli/developmental stage of the host represents pressure for the associated microbiome, selecting only specific microbial groups associated with primary branches, receptacles, and aerocysts.

### Algae deciduous and holdfast and axis parts harbor different microbiomes

The number of ASVs unique to the algae microbiome and at the same time shared among all parts of algal thalli was low (50 ASVs). The number of unique ASVs shared among deciduous algae parts was even lower (6), with 17 ASVs unique to primary branches, 9 for aerocysts, and 1 for receptacles, suggesting some level of body part specificity. The microbial group that seems to exhibit the most specificity toward algae deciduous parts was Acidimicrobiia (12.3%–28.6%), which represents only a minor part of holdfast-associated communities (less than 3.1%) and less than 2% of ambient microbiota (Fig. 4A). Accordingly, Acidimicrobiia were previously associated with another genus of brown algae from the *Sargassaceae* family (23, 54). Moreover, in our data set, basically the entire community of Acidimicrobiia was affiliated with the *Microtrichaceae* family, which was previously shown to constitute the core microbiome of green, brown, and red macroalgal phycosphere (55). Another important member of specific algae deciduous parts microbiota seems to be the *Granulosicoccaceae* family, which was present in very low proportion (<0.1% of sequence proportion) in ambient microbiome samples in our study. In line with this, *Granulosicoccus* was recruited as the most abundant metagenome-assembled bacterial genome from kelp-associated microbial communities, where their role in vitamin B12 production was highlighted (56). On the other hand, holdfast and axis were associated with a very diverse Gammaproteobacterial community, likely contributing to higher alpha diversity recorded for this algae body part (Fig. 3A and 4B). Interestingly, other reports on *Cystoseira s.l*. microbiome from our study area did not detect Acidimicrobiia and *Granulosicoccaceae* (24, 25).

Some bacterial groups, like Flavobacteriales, represented an important part of the algae microbiome (10.9%–55.9%), with no preference for thalli part, yet exhibited less host specificity, as they were present also in ambient sediment and seawater samples. Accordingly, Flavobacteriales were also reported as one of the predominant orders composing microbiomes of *Ericaria crinita*, *G. barbata,* and *Cystoseira compressa* from the Adriatic Sea (23–25). Flavobacteriales are known for their capacity to degrade a wide range of algae-derived polysaccharides, with a strong preference for algae-associated lifestyle (57). In line with our results, the same study also reported a high relative abundance of *Rhodobacteraceae* associated with all algal parts. While Blažina et al. (24) also reported Rhizobiales as a non-negligible part of the algal microbiome, this order was only detected as part of holdfast and axis-associated microbiota in our data set. In our study, we were able to find some tissue-specific bacterial taxa, which were unique to some algae thalli parts, e.g., Polyangia, which are micro predators capable of lysing cells or other microorganisms and cellulose degradations, were exclusively associated with aerocysts (58). Exclusively associated with holdfast and axis were *Nitrososphaera*, Puniceispirillales, Kiloniellales (previously isolated from macroalgae), and Parvibaculales, likely contributing to higher alpha diversities of algae thalli part communities. However, many bacterial groups were shared among holdfast and axis and sediment samples, likely representing part of recruited communities, which, however, did not survive selective pressures during the development of deciduous parts (e.g., Cytophagales, Bacteroidales, Desulfobacteria, Desulfobulbia, Planctomycetes, Desulfobulbia).

Note the discrepancy between this study and other *Cystoseira s.l*. microbiome studies from the area likely results from major methodological differences and bioinformatic pipelines applied, which means that the comparison is not straightforward. First, different sets of primers to amplify different 16S regions were applied (V3-V4 region [59], in Blažina et al. [24], and V4-V5 region [29], this study), a different sequence approach was used (operational taxonomy unit—OTU in Blažina et al. [24] and Malfatti et al. [25] vs amplicon sequence variant—ASVs in this study), and different bioinformatic pipeline and reference database versions were employed.

### Conclusions and future perspectives

We provide the first insight into the potential differences in the structure of microbiomes of two morphologically distinct but genetically identical *G. barbata* populations in the coastal anthropogenically impacted marine ecosystem. Our preliminary results suggest some environmental forcing on the structure of the algae microbiome; however, further analysis of chemical, microbiological, and other environmental factors is needed. Our baseline study allows us to formulate the first hypothesis regarding influences of environmental selective pressure and algal intrinsic factors on microbiome composition that we would like to address in the future. We show that the *G. barbata* microbiome exhibits relatively low host specificity, which was, however, somehow higher for basal algal parts than for deciduous parts. Based on these data, we suggest the second hypothesis that part of the algae microbiome is recruited from adjacent sediment communities, which becomes more specialized during algae growth, either due to the biochemical composition of different host body parts, different mechanistic basis for host–microbiome association, and/or ambient factors in the water column. Descriptive studies are important to gain insights into the diversity of host-associated microorganisms. However, these studies do not disentangle the cause–effect relationships in which the effects of the microbiota on the host (and vice versa) can be distinguished (60). Therefore, our initial findings on the microbiome of canopy-forming Fucales in the Gulf of Trieste raise interesting questions to be addressed in future research regarding the specific role of these epiphytic bacteria in macroalgal settlement, growth, and reproduction.

## Data Availability

Sequencing data for this study have been deposited in the European Nucleotide Archive (ENA) at EMBL-EBI under accession number PRJEB60814. All other data will be made available on request.

## SUPPLEMENTAL MATERIAL

Supplemental Figures spectrum.02204-24-s0001.docx Figures S1-S7.

Supplemental Tables spectrum.02204-24-s0002.xlsx Tables S1-S7.

Table S8 spectrum.02204-24-s0003.csv Family-level abundance.

ASM does not own the copyrights to Supplemental Material that may be linked to, or accessed through, an article. The authors have granted ASM a non-exclusive, world-wide license to publish the Supplemental Material files. Please contact the corresponding author directly for reuse.
