# Supplementary material for: Diversity of the surface microbiome of canopy-forming brown macroalgae (Fucales) in the northern Adriatic
Source: Microbiol Spectr. 2025 Apr 16;13(6):e02204-24. doi: 10.1128/spectrum.02204-24 (PMC12131803; doi:10.1128/spectrum.02204-24)
Supplement: Supplemental Figures — Figures S1-S7. [file spectrum.02204-24-s0001.docx]

**Supplementary Figures**

**
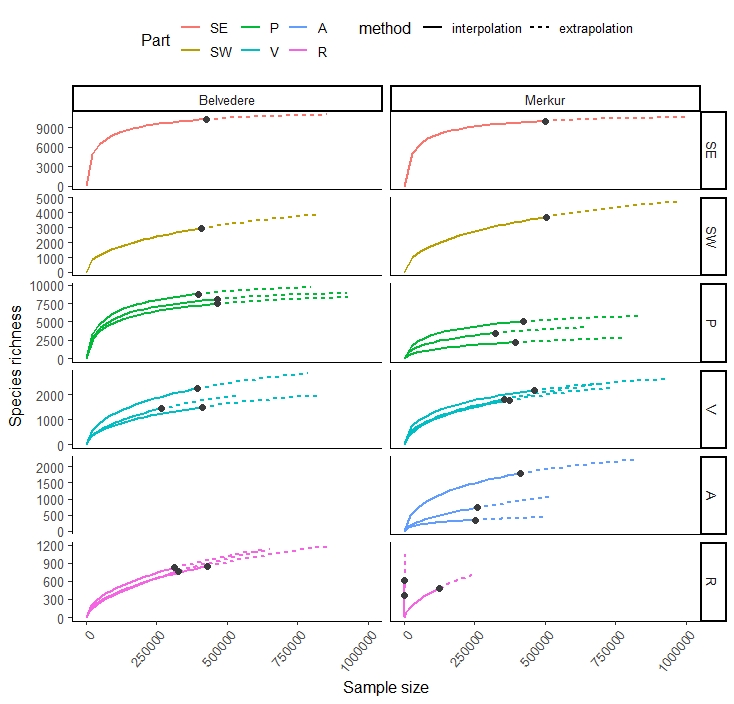
**

**Figure S1:** Rarefaction curves of 16S rRNA gene analysis of bacterial communities. The solid lines represent the observed accumulation with the number of reads sampled, and the dashed lines represent the extrapolated accumulation up to the double amount of reads. The observed values for each community are denoted by solid shapes. Sample-size-based rarefaction curves are generated with the R-package “iNEXT”, based on the Hill number of order q = 0. Please note that the y-axis values are not consistent across all graphs to ensure better readability.


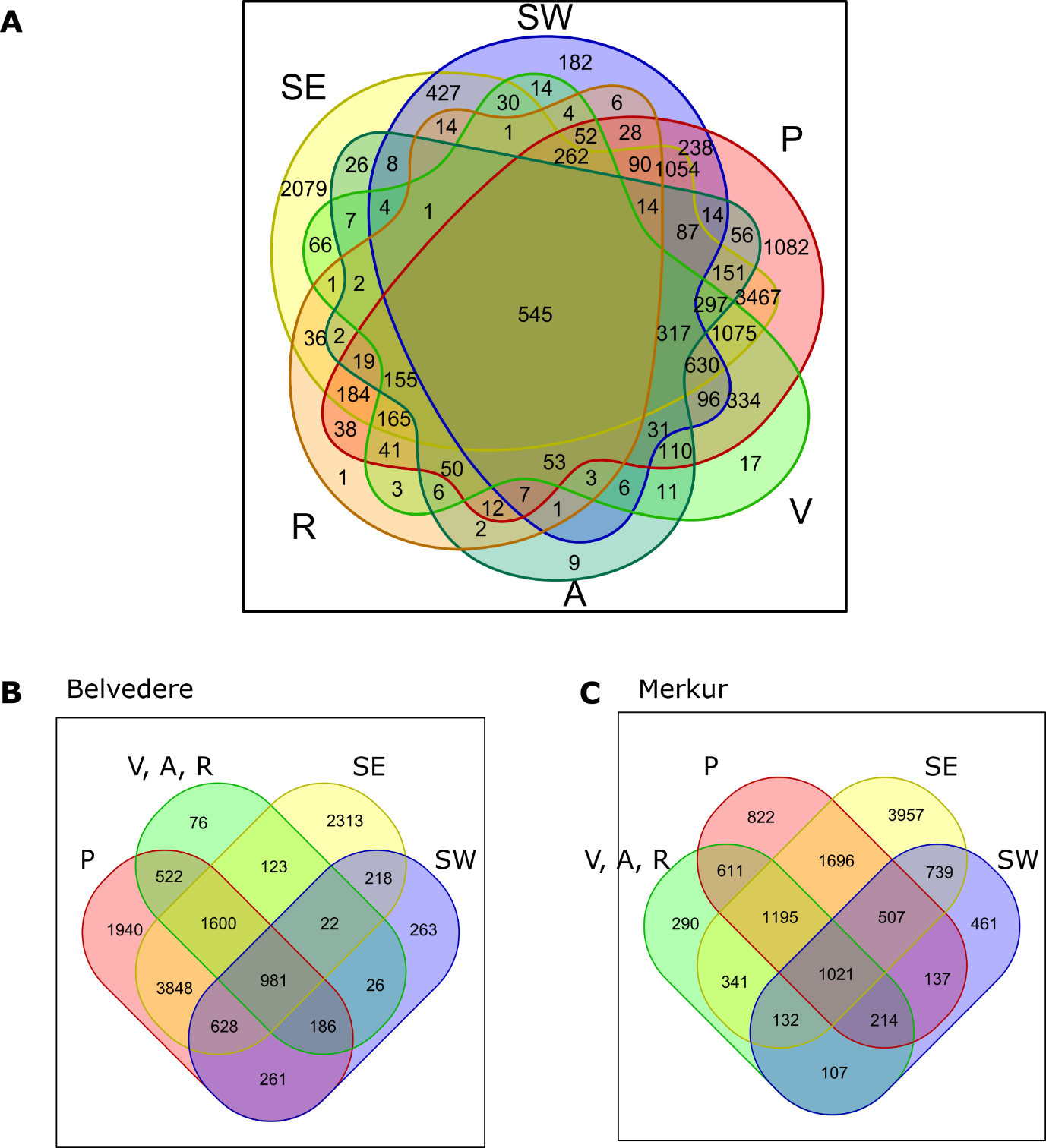


**Figure S2:** Visualization of bacterial community relationships between different samples based on the presence/absence of ASVs using a Venn diagrams. A - Relationship between different algal parts (P, V, A, R), ambient seawater (SW), and sediment (SE) in general. B, C - Relationship between different algal parts (deciduous parts i.e., V, A, R and holdfast and axis i.e., P), ambient seawater (SW), and sediment (SE), shown separately for the two locations. The location is indicated at the top of each Venn diagram for clarity.


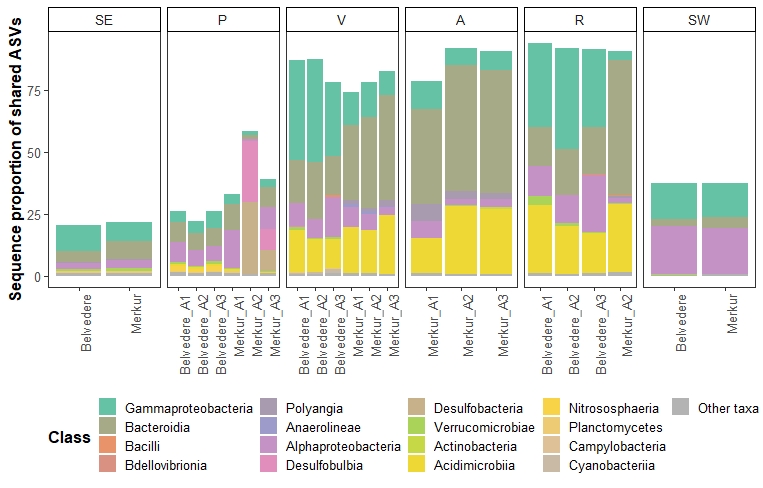


**Figure S3:** Sequence proportion of core microbiome ASVs (i.e., ASVs detected in all samples of our study set) in different algal parts (P, V, A, R), ambient seawater (SW), and sediment (SE). Classes showing a sequence proportion < 0.1% are grouped as “Other taxa”.


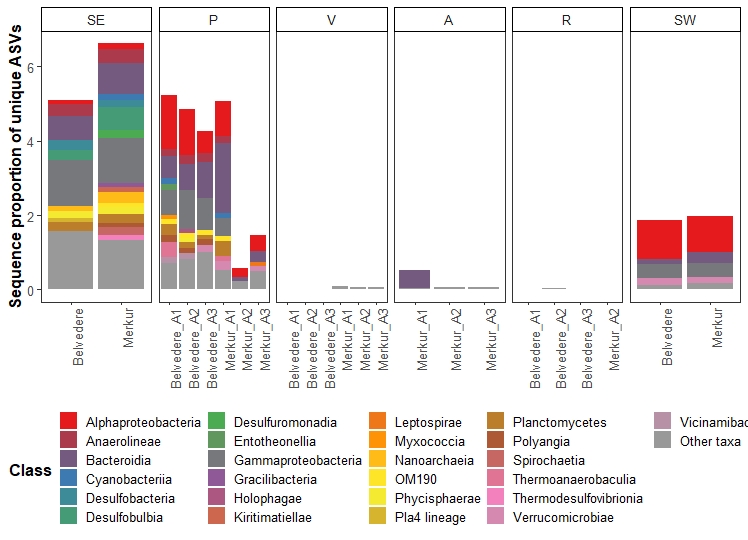


**Figure S4:** Sequence proportion of unique ASVs for different algal parts (P, V, A, R), ambient seawater (SW), and sediment (SE). Classes showing a sequence proportion < 0.1% are grouped as “Other taxa”.


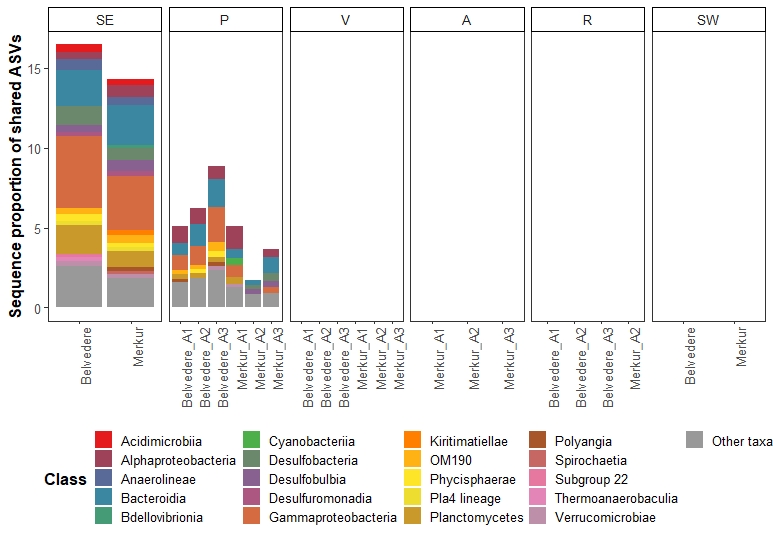


**Figure S5:** Sequence proportion of ASVs shared only between sediment and holdfast and axis (P) samples. Classes showing a sequence proportion < 0.2% are grouped as “Other taxa”.


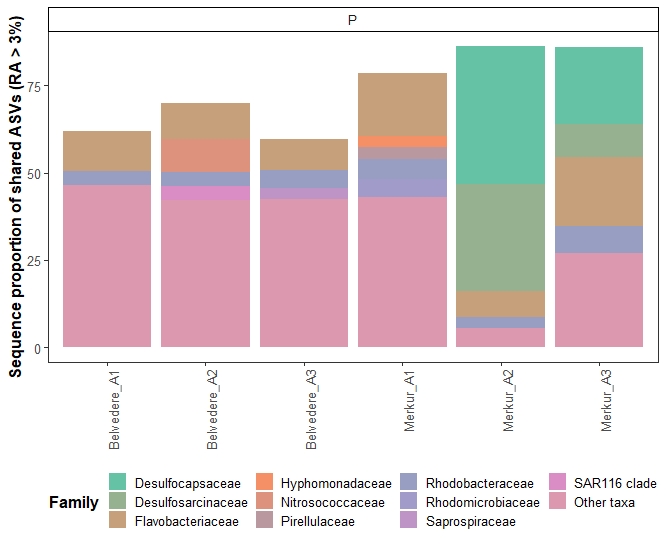


**Figure S6:** Shared P-associated ASVs between Belvedere and Merkur sampling station. Families showing a sequence proportion < 3% are grouped as “Other taxa”.


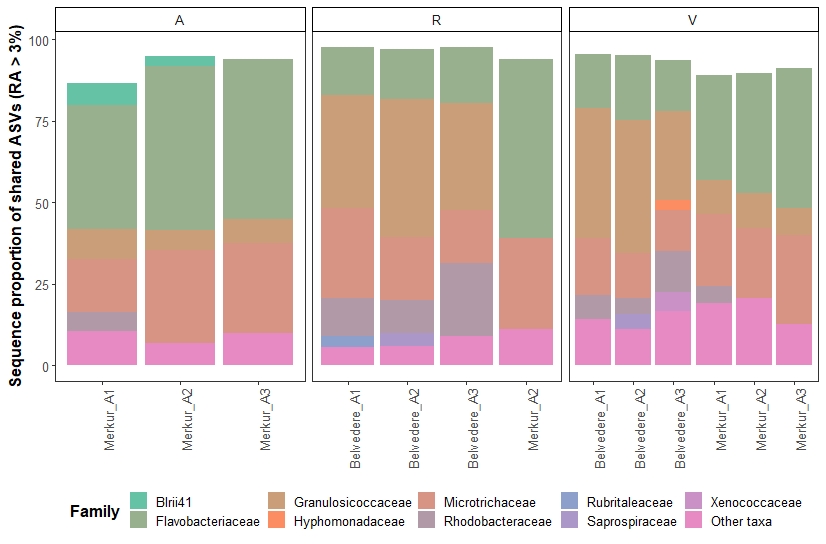


**Figure S7:** Shared algae associated ASVs (only deciduous part - A, R, V) between Belvedere and Merkur sampling station. Families showing a sequence proportion < 3% are grouped as “Other taxa”
